# TGF-β1, pSmad-2/3, Smad-7, and β-Catenin Are Augmented in the Pulmonary Arteries from Patients with Idiopathic Pulmonary Fibrosis (IPF): Role in Driving Endothelial-to-Mesenchymal Transition (EndMT)

**DOI:** 10.3390/jcm13041160

**Published:** 2024-02-19

**Authors:** Archana Vijay Gaikwad, Mathew Suji Eapen, Surajit Dey, Prem Bhattarai, Affan Mahmood Shahzad, Collin Chia, Jade Jaffar, Glen Westall, Darren Sutherland, Gurpreet Kaur Singhera, Tillie-Louise Hackett, Wenying Lu, Sukhwinder Singh Sohal

**Affiliations:** 1 Respiratory Translational Research Group, Department of Laboratory Medicine, School of Health Sciences, College of Health and Medicine, University of Tasmania, Launceston, TAS 7248, Australia; 2 National Health and Medical Research Council (NHMRC) Centre of Research Excellence (CRE) in Pulmonary Fibrosis, Respiratory Medicine and Sleep Unit, Royal Prince Alfred Hospital, Camperdown, NSW 2050, Australia; 3 Launceston Respiratory and Sleep Centre, Launceston, TAS 7250, Australia; 4 Department of Respiratory Medicine, Launceston General Hospital, Launceston, TAS 7250, Australia; 5 Department of Allergy, Immunology and Respiratory Medicine, The Alfred Hospital, Melbourne, VIC 3004, Australia; 6 Department of Immunology and Pathology, Monash University, Melbourne, VIC 3004, Australia; 7 Department of Anaesthesiology, Pharmacology and Therapeutics, University of British Columbia, Vancouver, BC V6T 1Z4, Canada; 8 Centre for Heart Lung Innovation, St. Paul’s Hospital, Vancouver, BC V6Z 1Y6, Canada

**Keywords:** idiopathic pulmonary fibrosis, pulmonary hypertension, pulmonary artery, vascular remodelling, endothelial-to-mesenchymal transition

## Abstract

**Background:** We have previously reported that endothelial-to-mesenchymal transition (EndMT) is an active process in patients with idiopathic pulmonary fibrosis (IPF) contributing to arterial remodelling. Here, we aim to quantify drivers of EndMT in IPF patients compared to normal controls (NCs). **Methods:** Lung resections from thirteen IPF patients and eleven NCs were immunohistochemically stained for EndMT drivers, including TGF-β1, pSmad-2/3, Smad-7, and β-catenin. Intima, media, and adventitia were analysed for expression of each EndMT driver in pulmonary arteries. Computer- and microscope-assisted Image ProPlus7.0 image analysis software was used for quantifications. **Results:** Significant TGF-β1, pSmad-2/3, Smad-7, and β-catenin expression was apparent across all arterial sizes in IPF (*p* < 0.05). Intimal TGF-β1, pSmad-2/3, Smad-7, and β-catenin were augmented in the arterial range of 100–1000 μm (*p* < 0.001) compared to NC. Intimal TGF-β1 and β-catenin percentage expression showed a strong correlation with the percentage expression of intimal vimentin (r′ = 0.54, *p* = 0.05 and r′ = 0.61, *p* = 0.02, respectively) and intimal N-cadherin (r′ = 0.62, *p* = 0.03 and r′ = 0.70, *p* = 0.001, respectively). Intimal TGF-β1 and β-catenin expression were significantly correlated with increased intimal thickness as well (r′ = 0.52, *p* = 0.04; r′ = 0.052, *p* = 0.04, respectively). Moreover, intimal TGF-β1 expression was also significantly associated with increased intimal elastin deposition (r′ = 0.79, *p* = 0.002). Furthermore, total TGF-β1 expression significantly impacted the percentage of DLCO (r′ = −0.61, *p* = 0.03). **Conclusions:** This is the first study to illustrate the involvement of active TGF-β/Smad-2/3-dependent and β-catenin-dependent Wnt signalling pathways in driving EndMT and resultant pulmonary arterial remodelling in patients with IPF. EndMT is a potential therapeutic target for vascular remodelling and fibrosis in general in patients with IPF.

## 1. Introduction

Idiopathic pulmonary fibrosis (IPF) is a chronic, highly progressive fibrotic lung disease with limited therapeutic options [1]. In IPF, abnormal interactions between the damaged epithelium and fibroblasts affect pathological lesions known as fibroblast foci, which are formed with activated myofibroblasts [2]. Activated myofibroblasts are the major cells responsible for the production, secretion, and remodelling of the extracellular matrix in IPF [3]. The abnormal extracellular matrix (ECM) deposition and damaged alveolar architecture result in poor gas exchange [1]. Moreover, aberrant ECM disposition in pulmonary vasculature leads to abnormal vascular remodelling and further disrupted gas exchange in IPF [4]. The origin of lung fibroblasts and their transformation into myofibroblasts plays a crucial role in the progression of pulmonary fibrosis [5,6]. Epithelial-to-mesenchymal transition (EMT) is well known as a source of fibroblasts in IPF [7]. Our recent findings have demonstrated that endothelial-to-mesenchymal transition (EndMT) could also be another source for myofibroblasts in IPF and may contribute to vascular remodelling or fibrosis in general [4].

Similar to EMT, EndMT is a mechanism whereby endothelial cells undergo molecular changes and gradually lose their cell-to-cell junctional properties, such as vascular endothelial cadherin (VE-cadherin), and gain mesenchymal proteins collagens, S100A4, vimentin, and α-smooth muscle actin (α-SMA) [8]. Different signalling pathways regulate the EndMT process, such as transforming growth factor-beta (TGF-β), Hedgehog (Hh), Wingless/intergrase-1 (WNT), and Notch and nuclear factor kappa B (NF-kB) signalling pathways [9]. The growth factors, including TGF-β, platelet-derived growth factor (PDGF), endothelin-1 (ET-1), and β-catenin, have been identified as major drivers of EndMT in other disease models such pulmonary hypertension or cardiac fibrosis [9,10]. However, the role of various EndMT drivers and signalling pathways in IPF is not fully understood yet.

Therefore, in this study, we quantitatively assessed EndMT drivers such as TGF-β1, pSmad-2/3, Smad-7, and β-catenin, in the classified pulmonary arteries from patients with IPF and compared them to normal controls (NCs). We also studied the correlation between the expression of these drivers in each arterial layer and EndMT markers such as endothelial junctional marker VE-cadherin and mesenchymal proteins vimentin, S100A4, and N-cadherin. Furthermore, we also analysed the correlation between the EndMT drivers’ expression and lung physiological parameters, such as the low diffusing capacity of the lungs for carbon monoxide (DLCO) in patients with IPF.

## 2. Materials and Methods

### 2.1. Study Population

We had access to resected IPF tissue from thirteen patients with IPF from The Alfred Health Biobank in Melbourne (Alfred Health Biobank Melbourne, ethics ID: 336-13). The pathologist verified all patients’ histology results for usual interstitial pneumonia. None of the patients was on antifibrotic medications. The James Hogg Lung Registry (ethics ID: H00-50110) at the University of British Columbia provided lung tissue from eleven NCs, who died due to reasons other than lung diseases. Lung function assessments were carried out by respiratory physicians. Detailed subject demographic information is provided in Table 1.

### 2.2. Immunohistochemical Staining for EndMT Driver Markers (TGF-β1, pSmad-2/3, Smad-7, and β-Catenin)

The immunohistochemistry method was used for all EndMT drivers. Briefly, lung tissue sections were deparaffinised in xylene and ethanol, respectively, followed by antigen retrieval performed at 110 °C for 15 min using target retrieval citrate buffer pH 6.0 (Dako S2369, Mulgrave, VIC, Australia) in a decloaking chamber (Biocare Medical, Pacheco, CA, USA). Primary antibodies were used to stain tissues for 60 min: mouse monoclonal TGF-β1 (1:2000, Abcam ab27969, Melbourne, VIC, Australia), polyclonal phospho-Smad2/Smad3 (pSmad-2/3, 1:100, ThermoFisher PA5-110155, Scoresby, VIC, Australia), mouse monoclonal Smad-7 (1:50, Santa Cruz Biotechnology sc-101152, Dallas, TX, USA), and rabbit monoclonal anti-β-catenin (1:100, Abcam ab32572, Melbourne, VIC, Australia), followed by secondary HRP rabbit/mouse antibodies (Dako K5007, Mulgrave, VIC, Australia). The DAB substrate (Dako K5007, Mulgrave, VIC, Australia) was added to make the protein markers visible, and hematoxylin (Australian Biostain P/L, Traralgon, Australia) was used to counterstain the nucleus. We have previously published using these techniques [4,11,12,13,14,15,16,17].

### 2.3. Measurement Strategies for Pulmonary Arteries’ Classification

We used 4× objective magnification to acquire images of the arteries from IPF patients and NC in a vertical uni-direction to eliminate overlap. We analysed samples using a Leica DM 500 microscope (Leica, Macquarie Park, NSW, Australia) with a Leica IC50W digital camera (Leica, Macquarie Park, NSW, Australia). The exterior length (from one end of the adventitia to the other) of pulmonary arteries was measured using ProPlus7.0 (Media cybernetics, Rockville, MD, USA) measuring tools. These measurements were utilized for arterial classification into six groups: 100–1000 µm (interspaced by 100 or 200 µm), identical to the approach employed in our previous studies [4,14].

### 2.4. Pulmonary EndMT Drivers’ (TGF-β1, pSmad-2/3, Smad-7, and β-Catenin) Total Arterial and Individual Layer Expression Measurement

We used different magnifications to acquire images of arteries with different sizes, such as for smaller arteries of 100–199 μm, 63× objective; for 200–399 μm and 400–599 μm, 40× objective; and for larger arteries of 600–1000 μm, 20× objective, respectively. All artery sizes were imaged without overlap, and five images per arterial size per individual were selected randomly using an online random number generator. For total arterial EndMT driver expression, Image ProPlus 7.0 software was used to manually choose an area of interest (AOI) from the beginning of the outer end of the intima towards the lumen to the adventitia outer border. For EndMT driver expression in individual layers, the AOI for each layer was also manually drawn using the same imaging software. Among individual layers, the intimal layer was selected from the outer luminal to the inner elastin layer; the medial layer was selected from the external layer of the inner elastin membrane to the internal lining of the external elastin membrane; and the adventitial layer was selected from the media’s exterior elastin layer to the arteries’ outermost connective tissue. Quantification of the expression of each EndMT driver in each arterial layer was counted as the total dark objects in the AOI, followed by counting the positive biomarker expression (in brown). The percentage EndMT driver expression was calculated using the following formula.

$$Each\;layer\;percentage\;of\;EndMT\;drivers’\;expression=\left.\left(\;\frac{number\;of\;selected\;colour\;objects\;in\;the\;AOI}{total\;number\;of\;the\;dark\;object\;in\;the\;AOI}\right.\right)\times 100$$

### 2.5. Correlations between the Mesenchymal Marker Expression and Vascular Remodelling Changes

Our group has previously published a study on the morphometric evaluation of vascular remodelling modifications observed in IPF patients [4,14]. We used the morphometric assessment data for correlations, as the patient cohort was identical. To determine the influence of EndMT drivers, the correlation between EndMT drivers’ and mesenchymal markers’ expression, as well as vascular remodelling changes such as arterial thickness, were assessed.

### 2.6. Statistical Analysis

The cross-sectional data presented in the study were tested for normality using the D’Agostino–Pearson omnibus normality test. We performed analyses of variance using ordinary one-way ANOVA and Bonferroni multiple comparison tests, which compare mean and standard deviation across all the groups of interest; specific group differences with correction for multiple comparisons were assessed using Dunn’s test. Regression analysis was performed using Pearman’s rank test. We used GraphPad prismV9 for all our analysis, with a *p*-value ≤ 0.05 considered significant.

## 3. Results

### 3.1. Morphological Assessment of EndMT Drivers’ Expression among Pulmonary Arteries

**Figure 1:**
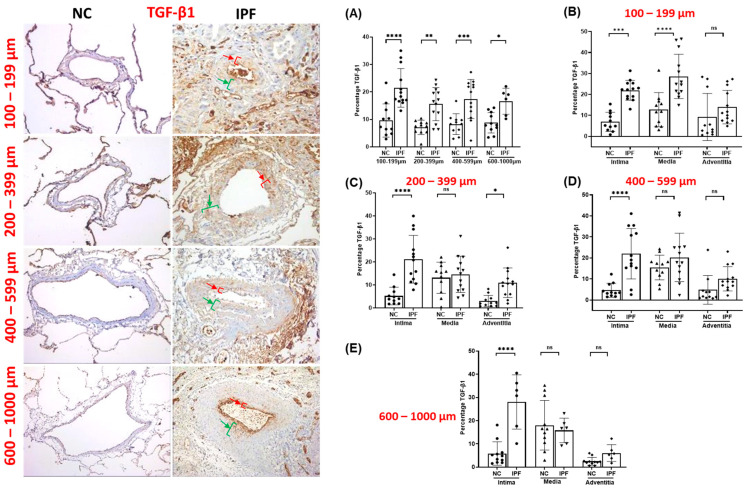
Immunohistology stained slides for pulmonary arteries from NC and IPF for TGF-β1: 100–599 µm (20× magnification) and 600–1000 µm (10× magnification). The red arrow indicates the intimal layer, and the green arrow indicates the median layer. Percentage of the positive expression of TGF-β1 (brown colour) in NC and IPF: all arterial sizes of (**A**) 100–1000 µm, and individual layers in different arterial sizes of (**B**) 100–199 µm, (**C**) 200–399 µm, (**D**) 400–599 µm, and (**E**) 600–1000 µm. The percentage expression was calculated using the formula in the method section. All data are presented as multiple comparisons with ordinary one-way ANOVA; ns = not significant, significance * *p* < 0.05, ** *p* < 0.01, *** *p* < 0.005, **** *p* < 0.001.

**Figure 2:**
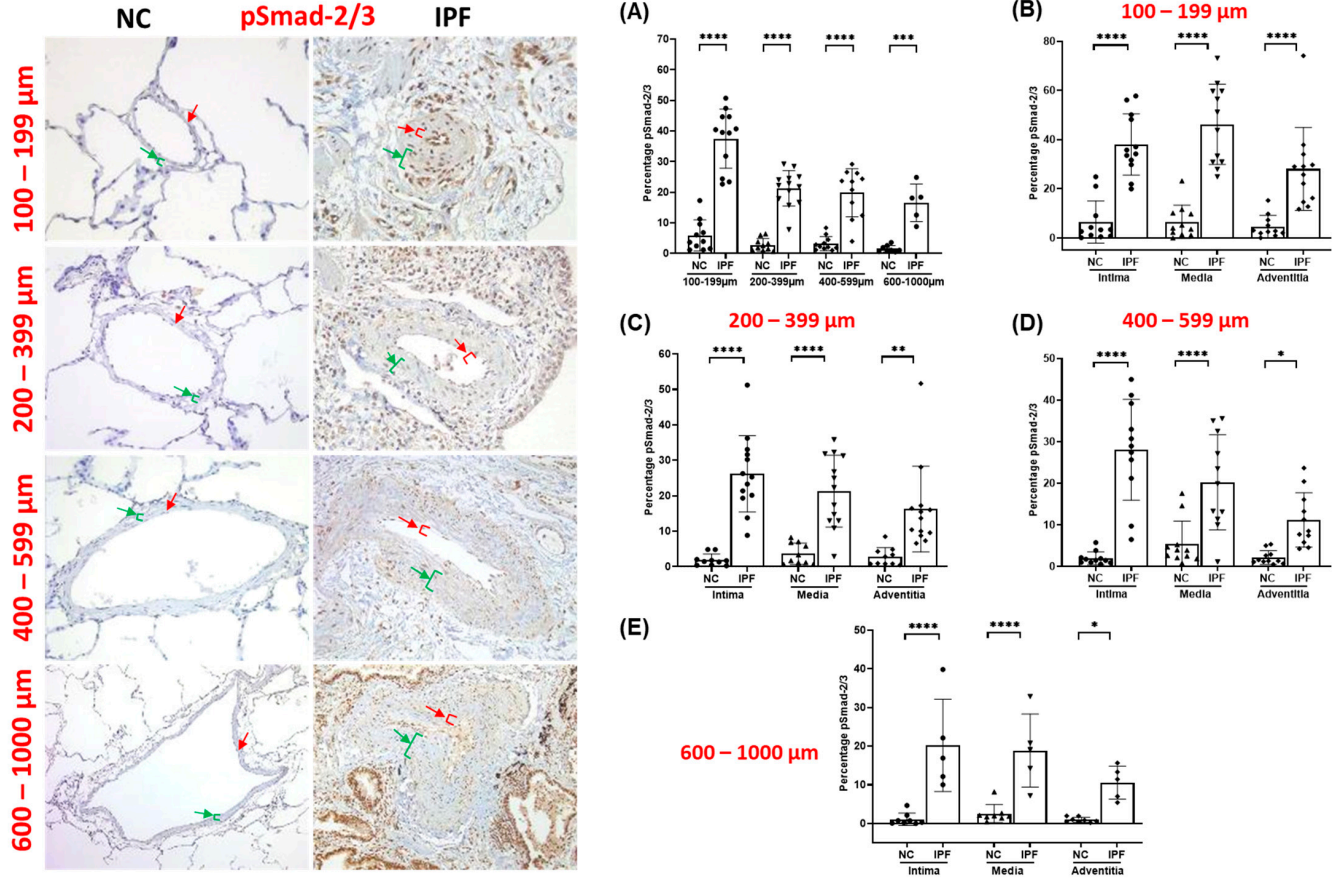
Immunohistology stained slides for pulmonary arteries from NC and IPF for pSmad-2/3: 100–599 µm (20× magnification) and 600–1000 µm (10× magnification). The red arrow indicates the intimal layer, and the green arrow indicates the median layer. Percentage of the positive expression of pSmad-2/3 (brown colour) in NC and IPF: all arterial sizes of (**A**) 100–1000 µm, and individual layers in different arterial sizes of (**B**) 100–199 µm, (**C**) 200–399 µm, (**D**) 400–599 µm, and (**E**) 600–1000 µm. The percentage expression was calculated using the formula in the method section. All data are presented as multiple comparisons with ordinary one-way ANOVA; significance * *p* < 0.05, ** *p* < 0.01, *** *p* < 0.005, **** *p* < 0.001.

**Figure 3:**
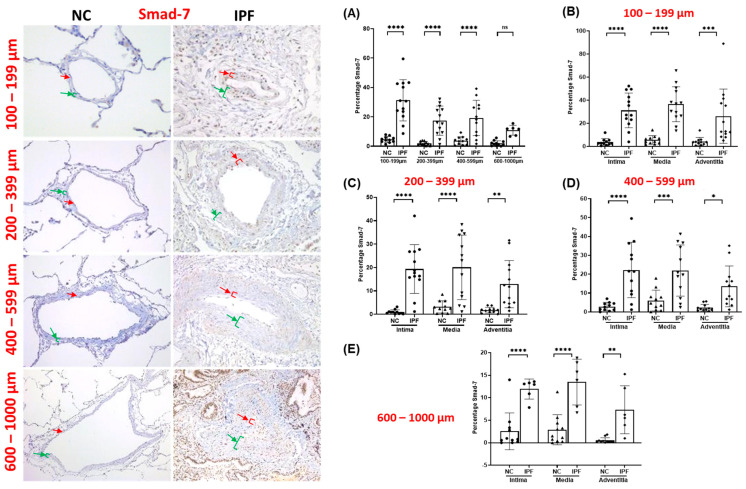
Immunohistology stained slides for pulmonary arteries from NC and IPF for Smad-7: 100–599 µm (20× magnification) and 600–1000 µm (10× magnification). The red arrow indicates the intimal layer, and the green arrow indicates the median layer. Percentage of the positive expression of Smad-7 (brown colour) in NC and IPF: all arterial sizes of (**A**) 100–1000 µm, and individual layers in different arterial sizes of (**B**) 100–199 µm, (**C**) 200–399 µm, (**D**) 400–599 µm, and (**E**) 600–1000 µm. The percentage expression was calculated using the formula in the method section. All data are presented as multiple comparisons with ordinary one-way ANOVA; ns = not significant, significance * *p* < 0.05, ** *p* < 0.01, *** *p* < 0.005, **** *p* < 0.001.

**Figure 4:**
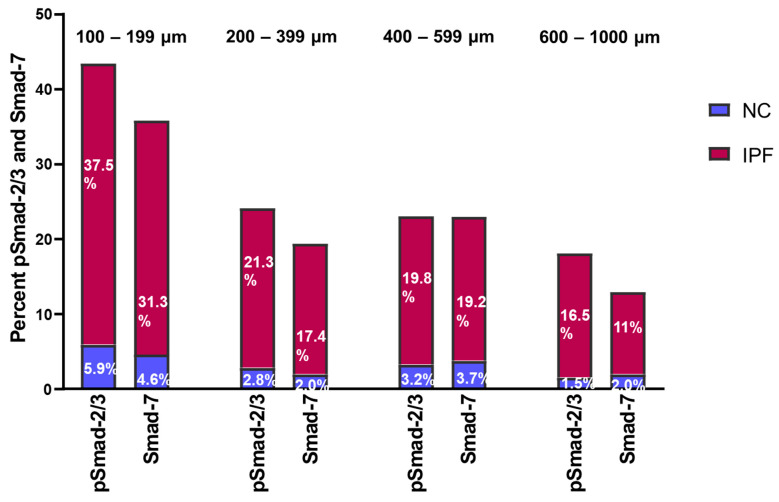
Fold change between pSmad-2/3 and Smad-7 in pulmonary arteries from NC and IPF.

The arteries from IPF patients displayed significant morphological alterations, including endothelial proliferation towards the lumen, proliferative intima, muscular hypertrophy of the intima and medial layer, and plexiform lesions, as compared to the arteries of NCs. Compared to NCs, TGF-β1, pSmad-2/3, and Smad-7 were highly expressed in the intimal, medial, and adventitia layers of classified arteries across all arterial ranges in IPF (Figure 1, Figure 2 and Figure 3). Additionally, β-catenin was extensively expressed in the intima, medium, and adventitia layers of arteries in IPF versus NCs (Figure 4). However, TGF-β1 expression was typically higher than β-catenin expression across all arterial ranges among IPF.

### 3.2. TGF-β1 Expression in Pulmonary Arteries

The percentage TGF-β1 expression increased significantly throughout all arterial sizes in IPF compared to NCs. TGF-β1 percentage was two-fold higher in IPF compared to NCs across all arterial sizes (*p* < 0.05) (Figure 1A). Compared to NCs, TGF-β1 percentage in intima was significantly greater (100–1000 µm (*p* < 0.001)), whereas media and adventitia did not indicate a comparable pattern across all arterial sizes in IPF (Figure 1B–E). TGF-β1 percentage was considerably greater in intima and media layers compared to adventitia in smaller-sized arteries of 100–199 µm (*p* < 0.001) (Figure 1B), and TGF-β1 percentage was significantly higher in intima (*p* < 0.0001) and adventitia (*p* < 0.05) compared to medial layer in medium-sized arteries of 200–399 µm in IPF patients (Figure 1C). Interestingly, TGF-β1 percentage was significantly higher in intima in the arterial range of 400–1000 µm (*p* < 0.0001) compared to medial and adventitia in IPF (Figure 1D,E). Clearly, the increasing trend in intimal TGF-β1 percentage across classified arteries indicates accelerated EndMT development via the TGF-β-dependent pathway in IPF.

### 3.3. pSmad-2/3 Expression in Pulmonary Arteries

The overall pSmad-2/3 percentage was considerably higher in IPF regardless of arterial sizes. Compared to NCs, total pSmad-2/3 percentage was much higher in smaller and medium-sized arteries in IPF (100–199 µm (*p* < 0.0001) and 200–399 µm (*p* < 0.0001)) (Figure 2A). Moreover, total pSmad-2/3 percentage was also significant in larger arteries (400–599 µm (*p* < 0.0001) and 600–1000 µm (*p* < 0.001)) (Figure 2A). Individual arteries also displayed greater fold changes in pSmad-2/3 percentage expression in IPF compared to NC, which indicates that the pSmad-2/3-dependent mesenchymal transition pathway is active in IPF. Intimal and medial pSmad-2/3 percentage expression was highly significant (*p* < 0.0001) compared to adventitia layers across all arterial sizes (100–1000 µm) (*p* < 0.5) (Figure 2B–E). Adventitial pSmad-2/3 percentage expression was significant among smaller (100–199 µm) (*p* < 0.0001) and medium (200–399 µm) (*p* < 0.0001) arteries (Figure 2B). Furthermore, adventitial pSmad-2/3 was also highly significant in the arterial range of 400–599 µm (*p* < 0.0001) (Figure 2D) compared to 600–1000 µm (*p* < 0.5) (Figure 2E).

### 3.4. Smad-7 Expression in Pulmonary Arteries

Compared to NCs, the total percentage of Smad-7 was considerably elevated throughout arterial ranges of 100–599 µm (*p* < 0.0001), except for larger arteries of 600–1000 µm (Figure 3A). The trend for Smad-7 percentage expression demonstrated substantial fold changes in the intima, media, and adventitial layers of classified arteries. Interestingly, intimal, and medial Smad-7 percentage expression displayed a similar pSmad-2/3 expression trend across all arterial ranges. In IPF, Smad-7 percentage in the intima and media layer was much higher throughout all arterial ranges, i.e., 100–1000 µm (*p* < 0.0001) and (*p* < 0.001), respectively (Figure 3B–E). Adventitial Smad-7 percent was also found to be increased across all arterial sizes, with significant expression in smaller arteries of 100–199 µm (*p* < 0.001) (Figure 3B) compared to medium arteries of 200–399 µm (*p* < 0.01) (Figure 3C). Nevertheless, larger arteries also displayed an enhanced adventitial Smad-7 percentage expression trend in the 400–599 µm (*p* < 0.05) (Figure 3D) and 600–1000 µm (*p* < 0.01) arterial range (Figure 3E).

### 3.5. Fold Change in pSmad-2/3 and Smad-7 Expression in Pulmonary Arteries

We also investigated the fold change in pSmad-2/3 and Smad-7 percentage in IPF and NCs. The overall fold change in pSmad2/3: Smad-7 increased proportionally, and on average, it was noted to be 1:1.2 among IPF and NCs (Figure 4). Compared to medium and larger arteries, the percentage expression of pSmad-2/3 and Smad-7 activity was highest in smaller arteries from IPF patients. The expression of pSmad2/3: Smad-7 in smaller arteries of 100–199 µm was 37.5%:31.3% in IPF and 5.9%:4.6% in NCs, whereas in medium arteries of 200–399 µm, it was 21.3%:17.4% in IPF and 2.8%:2% in NCs. In larger arteries of 400–599 µm, pSmad2/3: Smad-7 expression was 19.2%:19.8% in IPF and 3.2%:3.7% in NC, and in 600–1000 µm arteries, it was 16.5%:11.0% in IPF and 1.5%:2.0% in NC. Overall, this significant expression of pSmad2/3 and Smad-7 suggests that Smad-7 is unable to suppress the phosphorylation of Smad2/3 in IPF compared to NC.

### 3.6. β-Catenin Expression in Pulmonary Arteries

**Figure 5:**
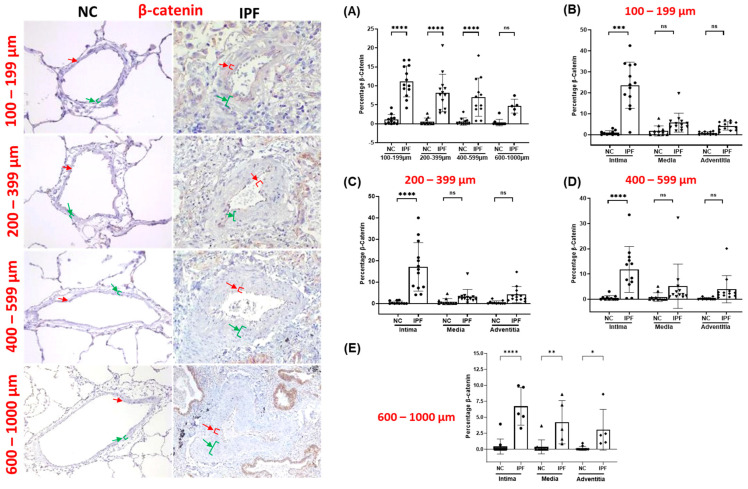
Immunohistology stained slides for pulmonary arteries from NC and IPF for β-catenin: 100–599 µm (20× magnification) and 600–1000 µm (10× magnification). The red arrow indicates the intimal layer, and the green arrow indicates the median layer. Percentage of the positive expression of β-catenin (brown colour) in NC and IPF: all arterial sizes of (**A**) 100–1000 µm, and individual layers in different arterial sizes of (**B**) 100–199 µm, (**C**) 200–399 µm, (**D**) 400–599 µm, and (**E**) 600–1000 µm. The percentage expression was calculated using the formula in the method section. All data are presented as multiple comparisons with ordinary one-way ANOVA; ns = not significant, significance * *p* < 0.05, ** *p* < 0.01, *** *p* < 0.005, **** *p* < 0.001.

Compared to NC, the total percentage of β-catenin was significantly elevated in all arterial ranges of 100–599 µm (*p* < 0.0001), apart from arteries of 600–1000 µm in IPF (Figure 5A). Total β-catenin percentage expression in larger arteries was two-fold greater in IPF than in NCs (Figure 5A), although the *p*-value is not significant statistically. In IPF, intimal β-catenin percentage was substantially increased throughout all arterial sizes of 100–1000 µm (*p* < 0.0001) (Figure 5B–E). This trend in intimal β-catenin percentage expression was found to match intimal TGF-β1 percentage expression. The medial and adventitial β-catenin percentage was not significantly higher than the intimal percentage across all arterial ranges (Figure 5B–D), except for larger arteries of 600–1000 µm (*p* < 0.05) in IPF (Figure 5E). In IPF, the overall trend of elevated intimal β-catenin expression across classified arteries suggests enhanced EndMT activation via the β-catenin-dependent WNT pathway.

### 3.7. Correlation between EndMT Drivers’ Expression and EndMT Markers

We assessed the correlations between the EndMT driver percentage expression and mesenchymal marker percentage expression and discovered a strong and consistent relationship among both. Total TGF-β1 expression percentage exhibited strong associations with increased total vimentin (r′ = 0.65, *p* = 0.02) and total N-cadherin (r′ = 0.69, *p* = 0.01) (Table 2, A and C). Interestingly, the total β-catenin percentage also displayed a strong association with the increased total percentage expression of vimentin (r′ = 0.53, *p* = 0.03) and N-cadherin (r′ = 058, *p* = 0.03) noted among IPF patients (Table 2, G and I). Additionally, we also found a similar positive relation between surged intimal TGF-β1 and β-catenin percentage expression and elevated intimal mesenchymal percentage expression in IPF. Intimal TGF-β1 percentage correlated strongly with increased intimal vimentin percentage (r′ = 054, *p* = 0.05) (Table 2, B) and intimal N-cadherin percentage (r′ = 0.62, *p* = 0.03) (Table 2, D), whereas elevated intimal β-catenin percentage also showed a strong association with elevation in intimal vimentin (r′ = 061, *p* = 0.02) (Table 2, H) and N-cadherin percentage expression (r′ = 0.70, *p* = 0.001) (Table 2, J). Nevertheless, we did not find such association between EndMT driver and mesenchymal protein S100A4 expression.

We previously reported that EndMT plays a key role in vascular remodelling in IPF. The endothelial marker VE-cadherin is translocated from the junction to the cytoplasm, which leads the endothelial cells to lose the junctional connection and obtain the mesenchymal features [14]. In this study, we also found increased expression for N-cadherin, vimentin, and S100A4 in the arterial layers, confirming EndMT. Therefore, we explored the association between percent intimal EndMT driver expression and endothelial protein VE-cadherin expression. Intimal TGF-β1 percentage correlated significantly and displayed a positive relationship with VE-cadherin junctional-positive cell percentage (r′ = 0.059, *p* = 0.03) (Table 2, E) and VE-cadherin cytoplasm-positive cell percentage (r′ = 0.61, *p* = 0.04) (Table 2, F); such a strong correlation could be the result of TGF-β’s dual influences on cell growth.

In contrast, there were contrasting associations observed between the percentage of β-catenin in the intimal layer and the presence of VE-cadherin-positive cells at junctions and in the cytoplasm. Specifically, the intimal percentage of β-catenin showed a negative correlation with the percentage of VE-cadherin-positive cells at junctions (r′ = −0.44, *p* = 0.09) (Table 2, K). This suggests that as the percentage of β-catenin in the intimal layer increases, there is a tendency for a decrease in the percentage of VE-cadherin-positive cells at junctions. However, it is important to note that the statistical significance of this association is not strong. On the other hand, the intimal percentage of β-catenin demonstrated a positive correlation with the percentage of VE-cadherin-positive cells in the cytoplasm (r′ = 0.70, *p* = 0.02), as shown in Table 2, L. This implies that as the percentage of β-catenin in the intimal layer increases, there is a tendency towards an increase in the percentage of VE-cadherin-positive cells in the cytoplasm. This correlation is statistically significant, indicating a stronger relationship. Based on these findings, it can be inferred that the higher levels of β-catenin in the intimal layer could play a role in the cellular changes associated with the loss of VE-cadherin at cell junctions in IPF.

### 3.8. Correlation between EndMT Drivers’ Expression and Vascular Remodelling Changes

According to the correlations between EndMT driver and vascular remodelling, it appears that TGF-β1 and β-catenin might be accountable for EndMT-related vascular remodelling observed among IPF patients. Total TGF-β1 percentage correlated strongly with increased total arterial thickness (r′ = 0.52, *p* = 0.04) (Table 3, A) and total elastin deposition (r′ = 0.68, *p* = 0.01) (Table 3, C). Interestingly, intimal TGF-β1 percentage correlated significantly with increased intimal thickness (r′ = 0.52, *p* = 0.04) (Table 3, B) and intimal elastin deposition (r′ = 0.79, *p* = 0.002) (Table 3, D). Total β1-catenin percentage also correlates significantly with increased total arterial thickness (r′ = 0.56, *p* = 0.05) (Table 3, E), whereas intimal β-catenin percentage also showed a strong association with increased intimal thickness (r′ = 0.52, *p* = 0.04) (Table 3, F). In the case of elastin deposition, β-catenin did not show such a significant positive relationship as TGF-β1; however, it shows a trend of positive contribution in elastin deposition. Total β-catenin and intimal β-catenin percentage also showed a positive relationship trend with increased total elastin (r′ = 0.22, *p* = 0.25) (Table 3, G) and intimal elastin disposition (r′ = 0.03, *p* = 0.46) (Table 3, H), respectively [18,19].

### 3.9. EndMT Drivers’ Impacts on Lung Physiology

We also found that EndMT drivers negatively impact lung functionality elements such as percent DLCO. There was a significant negative relationship between total TGF-β1 percentage and the percentage of DLCO (r′ = −0.61, *p* = 0.03) (Table 3, I). Interestingly, intimal TGF-β1 percentage also influenced the percentage of DLCO, but the *p*-value was not significant (r′ = −0.37, *p* = 0.15) (Table 3, J). Furthermore, total β-catenin and intimal β-catenin percentage also showed a negative relationship with the percentage of DLCO ((r′ = −0.16, *p* = 0.31) (Table 3, K) and (r′ = −0.13, *p* = 0.35) (Table 3, L), respectively).

## 4. Discussion

This is the first study to describe a size-based quantitative analysis of EndMT drivers in the classified arteries of IPF patients. Significant findings include increased TGF-β1, pSmad-2/3, Smad-7, and β-catenin percentage expression throughout arterial ranges in IPF compared to NCs. The levels of TGF-β1, pSmad-2/3, Smad-7, and β-catenin percentage were particularly increased in intimal layers, indicating an active endothelium transition. Additionally, EndMT key drivers such as TGF-β1 and β-catenin appear to stimulate the proliferation of the mesenchymal protein population, which contributes to vascular remodelling changes such as increased arterial thickness and elastin deposition in IPF. Furthermore, TGF-β1 and β-catenin appear to have a detrimental impact on lung physiological indicators such as the percentage of DLCO. Our findings would improve scientific knowledge about the EndMT drivers’ role in commencing vascular remodelling in IPF via Smad-dependent TGF-β1 and β-catenin-dependent Wnt signalling EndMT pathways. Furthermore, this study implies that therapeutic options for vascular remodelling in IPF could be explored by targeting key triggers of EndMT.

The role of the TGF-β superfamily as a vital switch in the onset and progression of IPF has been well established [20,21,22,23,24,25]. Our study also confirmed the existence of increased TGF-β1 in the muscular arteries of IPF patients. Intriguingly, enhanced TGF-β1 expression was predominantly noticed in the intimal layer across the arterial range, which confirms the active endothelial transformation. Earlier, several researchers emphasized that TGF-β signalling is involved in myofibroblast development via EndMT [26,27,28,29]. Our study also found that augmented TGF-β1 expression substantially contributed to increased expression of mesenchymal protein vimentin and N-cadherin in IPF arteries, particularly in the intimal layer. Furthermore, this study discovered that TGF-β1 significantly contributed to vascular remodelling changes, such as increased total arterial thickness and intimal thickness. The TGF-β family has been believed to enhance myofibroblast development, accelerate the production and deposition of ECM proteins, and possibly trigger the mesenchymal transition of endothelial or other cell types in the pulmonary arteries [30,31,32]. Hence, our data imply that EndMT could be driving the remodelling changes through the activated TGF-β pathway in IPF arteries.

TGF-β induced EndMT is predominantly mediated by the phosphorylation of Smad proteins, which modulates the expression of important target genes [33,34,35]. The TGF-β/Smad-dependent signalling pathway is a crucial pathway in IPF pathophysiology [36,37]. Hence, we further investigated Smad family transcription factor′s role in pulmonary arteries from IPF patients and discovered significant augmented pSmad-2/3 expression in the intima, media, and adventitia layer across the arterial range. Roach et al. also noted elevated Smad-2/3 signalling in human lung myofibroblasts derived from IPF compared to non-fibrotic healthy control lungs [23]. Also, prolonged phosphorylation of Smad-2 in response to TGF-β was noted to be dependent on Smad-3 activity and essential for myofibroblast differentiation [38]. Furthermore, a cell model study also demonstrated that TGF-β1 stimulates dramatically elevated expression of Resistin-like molecule-β via the Smad-2/3/4 pathway, which has been shown to promote TGF-1-induced cell proliferation and EndMT [39]. Recent evidence suggests a signalling imbalance between the two primary canonical pathways in PH, with underactive Smad-1/5/8 signalling coexisting with overactive Smad-2/3 signalling in pulmonary arterial endothelial cells and smooth muscle cells [40]. Therefore, notable augmented intimal TGF-β1 and pSmad-2/3 expression across arterial sizes observed by our group also suggest that the Smad-2/3-dependent TGF-β/signalling pathway could be active in IPF arteries, as previously noted in chronic pulmonary vascular disease [41]. However, we observed that TGF-β1 is mostly upregulated in the intimal layer, but pSmad2/3 is expressed throughout the arterial layer. This suggests that there could be TGF-β1-independent mechanisms active in other arterial layers, which warrants further investigation [28,42].

Smad-7 is an inhibitory Smad in the TGF-β signalling pathway, and suppression of Smad-7 may lead to fibrosis [43,44]. Smad-7 was also noted as a crucial regulator of α-SMA and collagen I production in response to TGF-β1 [45]. Likewise, a significant anti-IPF effect of Smad-7 has been demonstrated in vitro and in vivo [46]. Furthermore, a recent animal study also showed that upregulating Smad-7 inhibits the TGF-β1/Smad signalling pathway and alleviates pulmonary fibrosis [47]. Notably, our investigation of the intima, media, and adventitia of IPF arteries revealed elevated Smad-7 expression comparable to pSmad-2/3. Moreover, our assessment of pSmad-2/3 and Smad-7-fold change revealed that both pSmad-2/3 and Smad-7 were increased proportionally in both NC and IPF. However, the percentages of both pSmad-2/3 and Smad-7 were exponentially amplified in IPF compared to NC. This result implies that Smad-7 could not impose the inhibitory effect on EndMT triggered by Smad-2/3-dependent TGF-β signalling, resulting in abundant myofibroblast development in IPF. Consequently, the TGF-β/Smad-dependent signalling pathway should be further investigated as a therapeutic target for the progression of IPF.

We further explored the role of β-catenin proteins in vascular remodelling observed in IPF. We discovered augmented β-catenin expression across all arterial sizes, significantly higher in the intima. However, we did not find such a significant expression in media and adventitia layers. Intimal augmented β-catenin expression indicates that β-catenin-dependent Wnt signalling potentially drives the vascular remodelling changes observed in IPF. Previously, overexpression of β-catenin proteins in the nucleus of IPF tissue sections suggested Wnt signalling might be activated in IPF patients [48]. In pulmonary endothelial cells, the activated canonical Wnt/β-catenin signalling pathway encourages perivascular fibroblasts to undergo a myofibroblast-like transformation, resulting in ECM deposition and increased tissue stiffness, which accelerates the progression of pulmonary fibrosis [49,50]. Furthermore, β-catenin expression in pulmonary artery endothelial cells was higher in individuals with idiopathic pulmonary arterial hypertension than in healthy controls [51]. Remarkably, we also noticed that total increased β-catenin contributed significantly to the increased expression of mesenchymal protein such as vimentin and N-cadherin in IPF; particularly, intimal β-catenin contributed to intimal increased myofibroblasts. Moreover, our study also revealed that β-catenin greatly contributed to vascular remodelling changes such as total arterial thickness and intimal thickness, potentially leading to PH. Recent evidence suggests that enhanced β-catenin signalling triggers the transition of endothelial cells to myofibroblasts [52] and promotes vascular remodelling in PH [53,54]. Evidence suggests that the canonical Wnt/β-catenin and TGF-β signalling pathways could functionally interact with each other [55,56,57,58]. However, further investigations are required to understand the cellular and molecular pathways of myofibroblast transformation in response to TGF- β and β-catenin in IPF pathophysiology [59,60,61].

Furthermore, our study discovered β-catenin expression was downregulated when endothelial cells were VE-cadherin junctional-positive. In contrast, β-catenin expression was upregulated when endothelial cells were VE-cadherin cytoplasmic-positive. The β-catenin is a multifunctional protein involved in Wnt signalling and cell–cell adhesion [62,63]. The expression of recombinant VE-cadherin cytoplasmic domain construct has been noted to compete with native VE-cadherin for β-catenin binding and improve the permeability of endothelial cell monolayers [64,65]. Thus, the positive association between β-catenin and VE-cytoplasmic-positive cells observed in this study could indicate cadherin-mediated cell adhesion loss, which could promote β-catenin signalling [66]. Furthermore, TGF-β1 expression was upregulated when endothelial cells were VE-cadherin junctional- and cytoplasmic-positive. This finding shows a possible link to TGF-β’s multifunctional role in regulating endothelial cell proliferation, differentiation, migration, and survival [67].

We further investigated the relationship between EndMT driver expression and lung function parameter DLCO percentage. The correlations confirm that the increased TGF-β1 and β-catenin expression impact lung gas exchange capacity (DLCO) in IPF patients, which could indicate underlying PH complications in IPF patients, as reduced DLCO in IPF patients has been believed to be a concomitant PH indication as to the underlying pulmonary fibrosis [68,69].

One of the limitations of this study is that the difference between the number of IPF and NC samples used for size-based comparisons varied, and certain artery sizes were not detected in IPF. A further limitation is the lack of cardiac function data or a clinical diagnosis of PH for the IPF patients investigated; hence, no relationships between EndMT drivers and PH could be substantiated. However, the positive association between EndMT drivers and vascular remodelling and the negative correlation between EndMT drivers and the percentage of DLCO implies the possibility of underlying PH in IPF.

In summary, this is the first study that provides a size-based quantification of EndMT drivers in IPF patients. Significantly increased TGF-β1, pSmad-2/3, Smad-7, and β-catenin expression indicate activated TGF-β/Smad-2/3-dependent signalling and β-catenin-dependent Wnt signalling pathways in pulmonary arteries. Increased TGF-β1 and β-catenin expression contribute to increased mesenchymal protein expression and arterial remodelling. Furthermore, augmented TGF-β1 and β-catenin expression impacted the gas exchange capacity of the lung. Hence, our study data validate the crucial role of EndMT drivers in IPF pathology and suggest targeting EndMT drivers as a therapeutic target [70] for vascular remodelling and underlying concomitant PH observed in IPF patients.

## Figures and Tables

**Table 1:** Subject demographics and clinical characteristics.

	Normal Control (NC)	IPF
Factor	Values *	
Total number (*n*)	11	13
Age	41 ± 15.1	64 ± 5.06
Gender (F/M)	6/5	6/7
Body mass index	NA	26.69 ± 3.03
Smoking status:		
Current, ex-smoker, and never (*n*)	Non-smokers	0/7/6
Smoking pack-years	-	20.84 ± 23.16
Lung physiology		
FEV_1_ (L)	NA	1.70 ± 0.40
FEV_1_ (%) predicted	NA	60.17 ± 12.22
FVC (L)	NA	1.97 ± 0.51
FVC (%) predicted	NA	53.5 ± 12.98
DLCO (mL/min/mmHg)	NA	5.91 ± 2.92
DLCO corrected (%) predicted	NA	25.85 ± 15.30

* Values represent the mean and the standard deviation; NA—not available.

**Table 2:** Correlation analysis—EndMT drivers with mesenchymal markers.

Correlation Analysis					
**TGF-β1 vs. EndMT Markers**					
(A) Total TGF-β1 vs. total vimentin	(B) Intimal TGF-β1 vs. intimal vimentin	(C) Total TGF-β1 vs. total N-cadherin	(D) Intimal TGF-β1 vs. intimal N-cadherin	(E) Intimal TGF-β1 vs. VE-cadherin junctional-positive	(F) Intimal TGF-β1 vs. VE-cadherin cytoplasm-positive
r′ = 0.65 *p* = 0.02	r′ = 0.54 *p* = 0.05	r′ = 0.69 *p* = 0.01	r′ = 0.62 *p* = 0.03	r′ = 0.59 *p* = 0.03	r′ = 0.61 *p* = 0.04
**β-Catenin vs. EndMT markers**					
(G) Total β-catenin vs. total vimentin	(H) Intimal β-catenin vs. intimal vimentin	(I) Total β-catenin vs. total N-cadherin	(J) Intimal β-catenin vs. intimal N-cadherin	(K) Intimal β-catenin vs. VE-cadherin junctional-positive	(L) Intimal β-catenin vs. VE-cadherin cytoplasm-positive
r′ = 0.53 *p* = 0.03	r′ = 0.61 *p* = 0.02	r′ = 0.58 *p* = 0.03	r′ = 0.70 *p* = 0.001	r′ = −0.44 *p* = 0.09	r′ = 0.70 *p* = 0.02

*p * <  0.05 was considered significant.

**Table 3:** Correlation—EndMT drivers and arterial thickness, elastin, and lung function.

Correlation Analysis			
TGF-β1 vs. Arterial thickness		TGF-β1 vs. Arterial elastin	
(A) Total TGF-β1 vs. total arterial thickness	(B) Intimal TGF-β1 vs. intimal thickness	(C) Total TGF-β1 vs. total arterial elastin	(D) Intimal TGF-β1 vs. intimal elastin
r′ = 0.52 *p * = 0.04	r′ = 0.52 *p * = 0.04	r′ = 0.68 *p * = 0.01	r′ = 0.79 *p * = 0.002
β-Catenin vs. Arterial thickness		β-Catenin vs. Arterial elastin	
(E) Total β-catenin vs. total arterial thickness	(F) Intimal β-catenin vs. intimal thickness	(G) Total β-catenin vs. total arterial elastin	(H) Intimal β-catenin vs. intimal elastin
r′ = 0.56 *p * = 0.05	r′ = 0.52 *p * = 0.04	r′ = 0.22 *p* = 0.25	r′ = 0.03 *p* = 0.46
TGF-β1 vs. DLCO (%)		β-Catenin vs. DLCO (%)	
(I) Total TGF-β1 vs. DLCO (%)	(J) Intimal β-catenin vs. DLCO (%)	(K) Total β-catenin vs. DLCO (%)	(L) Intimal β-catenin vs. DLCO (%)
r′ = −0.61 *p * = 0.03	r′ = −0.13 *p* = 0.35	r′ = −0.16 *p* = 0.31	r′ = −0.13 *p* = 0.35

*p*  <  0.05 was considered significant.

## Data Availability

All data are available upon request from the corresponding author.
